# High Levels of BCOX1 Expression Are Associated with Poor Prognosis in Patients with Invasive Ductal Carcinomas of the Breast

**DOI:** 10.1371/journal.pone.0086952

**Published:** 2014-01-28

**Authors:** Tong Liu, Xian-Yu Zhang, Xiao-Hui He, Jing-Shu Geng, Yang Liu, De-Jia Kong, Qing-Yu Shi, Feng Liu, Wei Wei, Da Pang

**Affiliations:** 1 Department of Breast Surgery, The Third Affiliated Hospital of Harbin Medical University, Harbin, China; 2 Department of Medical Records, The Third Affiliated Hospital of Harbin Medical University, Harbin, China; 3 Department of Pathology, The Affiliated Tumor Hospital of Harbin Medical University, Harbin, China; 4 Department of Medical Genetics, Harbin Medical University, Harbin, China; B.C. Cancer Agency, Canada

## Abstract

This study was to examine the breast cancer-overexpressed gene 1 (BCOX1) expression in invasive ductal carcinomas (IDC) of the breast and its value in the prognosis of the disease. The levels of BCOX1 expression in 491 paired IDC and surrounding non-tumor breast tissues as well as 40 paired fresh specimens were evaluated by tissue microarray, immunohistochemistry and quantitative RT-PCR. The potential associations of high BCOX1 expression with clinicopathological variables and the overall survival of these patients were analyzed. The relative levels of BCOX1 mRNA transcripts in the IDC breast tissues were significantly higher than that in the corresponding non-tumor tissues (P = 0.005). The anti-BCOX1 was predominantly stained in the cytoplasm of breast tissue cells and the levels of BCOX1 expression in the majority of breast cancer tissues were obviously higher than that in the corresponding non-tumor breast tissues. High levels of BCOX1 expression were found in 59.5% (292/491) of breast cancer tissues. The high BCOX1 expression was significantly associated with high histological grade (P = 0.037), positive expression of human epidermal growth factor receptor 2 (HER2, P = 0.031) and triple negative breast cancer (P = 0.027). The high BCOX1 expression in breast cancers was significantly associated with a shorter overall survival of these patients (P = 0.023), particularly in patients with triple negative breast cancer (P = 0.005). Therefore, the high BCOX1 expression may serve as a novel marker of poor prognosis and a potential therapeutic target for patients with IDC of the breast.

## Introduction

Breast cancer is the most common malignancy in women worldwide, and is the second leading cause of cancer-related mortality in women in the United States [1]. Despite improvements in cancer therapy, about one quarter of the patients diagnosed with invasive breast carcinomas (IDC) of the breast will eventually die from the disease. Identifying patients at a high risk of breast cancer may be helpful in the prevention of breast cancer and early diagnosis of patients with breast cancer is the best strategy for the protection against breast cancer-related morbidity and mortality. Therefore, discovery of effectively prognostic factors of breast cancer has become an important area of ongoing investigation [2], [3].

The breast cancer-overexpressed gene 1 (BCOX1) gene maps to chromosome 17q11.2 and was first reported in 2006 [4]. By screening SAGE and EST libraries, they identified 71 tags with specific expression in breast carcinomas. The BCOX1 matched the gene KIAA0100, which is a hypothetical gene by analyzing cDNA clones from human cell line KG-1 [5]. The BCOX1 appears to be an alternative splicer, in comparison with the KIAA0100-related sequences in humans and other species. The BCOX1 was predicted to encode a 222-amino acid BCOX1 protein, with an estimated molecular mass of 24.9 kD, containing a C-terminal tail unique among KIAA0100-related sequences. The BCOX1 mRNA transcripts were predominantly detected in the cytoplasm of breast cancer cells, particularly in malignant breast tissues, such as invasive and metastatic breast cancer cells. The BCOX1 mRNA transcripts were detected in non-tumor placenta and pancreas, but not in the normal breast tissues [4]. These data suggest that the BCOX1 expression may be associated with the development of breast cancer, particularly for invasive and metastatic breast cancer. However, there is no information about how the BCOX1 expression is associated with the clinicopathological features of IDC of the breast. Furthermore, it is unclear what factors are associated with the BCOX1 expression in breast cancer and what the value of BCOX1 expression is in the prognosis of patients with IDC of the breast.

In this study, the levels of BCOX1 expression in 40 paired IDC and corresponding non-tumor breast tissues freshly dissected and another 491 breast cancer tissue blocks were evaluated by quantitative real time polymerase chain reaction (qRT-PCR), tissue microarray and immunohistochemistry. The potential association of the levels of BCOX1 expression with the overall survival (OS) of patients was evaluated. The potential factors affecting the levels of BCOX1 expression were also analyzed in this population.

## Materials and Methods

### Patients and Tissue Specimens

A total 40 patients with IDC of the breast were subjected to surgical resection of breast cancer at the Third Affiliated Hospital and Tumor Hospital of Harbin Medical University. Their fresh breast cancer and corresponding non-tumor breast samples were collected, frozen in liquid nitrogen within 10 min immediately after surgical resection and stored at −80°C at the Heilongjiang Breast Tumor Biobank. Besides, 491 paired paraffin-embedded IDC and and corresponding non-tumor breast tissue blocks were obtained from the Department of Pathology of the Third Hospital of Harbin Medical University. The percentages of breast cancers in tumor specimens were >75%. Written informed consent was obtained from individual patients and the experimental protocol was approved by the Ethics Committee of Harbin Medical University.

The selected patients had a complete medical record from 2006. All patients had a pathological diagnosis of breast tissues and all samples were collected before any radiotherapy or chemotherapy. Individual samples were routinely tested for the expression of estrogen receptor (ER), progesterone receptor (PR), human epidermal growth factor receptor 2 (HER2), Ki-67 and P53 by immunohistochemistry. Individual samples with ≥ 14% of Ki67+ tumor cells were considered as high proliferation [6]. The intensity of anti-HER2 staining in all samples was semi-quantitatively analyzed and graded as 0–3. Individual samples with a grade of 0 or 1 were considered negative, samples with a grade of 2 as indeterminate and samples with a grade of 3 as positive. All samples were subjected to in duplicate fluorescence in situ hybridization (FISH) analysis. Samples with a <2-fold change in expression were regarded as negative, and those with a >2-fold increase were regarded as positive for the gene amplification [7], [8].

According to the Scarff-Bloom-Richardson (SBR) system, the invasiveness of cancers was determined by the frequency of cell mitosis, tubule formation, and nuclear pleomorphism, and classified as low (grade I), moderate (grade II), and high grades (grade III). Subsequently, individual cancers were further graded as negative (grade I) and positive (grade II and III). The presence of lymph node metastasis and tumor size (negative: ≤2 cm; positive: >2 cm) of individual patients were also recorded.

Individual breast cancers were subjected to molecular subtypes, according to the criteria as follows: Luminal A type: ER or/and PR positive, HER2 negative and Ki-67+ cells <14%; Luminal B type: (HER2 negative) ER or/and PR positive, HER2 negative and Ki-67+ cells ≥14%, (HER2 positive) ER or/and PR positive, and HER2 over-expressed or/and amplified; HER2 type: ER and PR negative and HER2 over-expressed or/and amplified; and triple negative breast cancer type: ER, PR, and HER2 negative [9].

All patients were followed up at least for five years at the Affiliated Hospital of Harbin Medical University and their clinical records were periodically reviewed. The period of OS in individual patients was defined as the time from the date of surgery to the date of first recurrence or death.

### Quantitative Real-time PCR (qRT-PCR)

Total RNA and DNA was extracted from fresh frozen samples using Trizol Reagent (Invitrogen; Carlsbad, CA), according to the manufacturer’s instructions. After quantified in a NanoDrop spectrophotometer (Thermo Scientific), 100 ng total RNA was reversely transcribed into cDNA using High-Capacity cDNA Reverse Transcriptase Kits (Applied Biosystems; Foster City, USA). The relative levels of target gene mRNA transcripts and DNA to the control GAPDH were determined by qRT-PCR using specific primers. The sequences of primers were forward 5′-GGTGGATCAAAAGGAACTGTC-3′ and reverse 5′-TTGGCTCAACTAAGTTTTCTGT-3′ for BCOX1 mRNA; forward 5′-TCTTGGCTGCCTTGTTCT-3′; reverse, 5′- ACTCAATGCTGGTTCTGC-3′ for BCOX1 DNA; forward 5′-GCCAGCCGAGCCACAT-3′ and reverse 5′-CTTTACCAGAGTTAAAAGCAGCCC-3′ for GAPDH. The PCR amplification was performed in triplicate at 95°C for 3 min and subjected to 40 cycles of 95°C for 15 sec, and 60°C for 40 sec. Negative controls consisted of distilled H_2_O. The relative levels of BCOX1 mRNA transcripts to the control GAPDH were determined by the 2^−ΔΔCt^ method.

### Tissue Microarray and Immunohistochemistry

At least two 3-mm tissue cores from the selected areas of individual paraffin-embedded tissue blocks were punched out using a punch machine and were placed into a recipient block. All IDC and corresponding non-tumor breast tissue blocks were cut to 4 µm with a microtome and arrayed in triplicate. Each tissue core was assigned a unique tissue microarray location number linked to a database containing other clinicopathological data.

The tissue sections were dried at 70°C for 3 hrs. After deparaffinization and rehydration, the sections were washed in phosphate-buffered saline (PBS; 3×3 min) and were treated with 3% H_2_O_2_ in methanol in the dark for 5–20 min. After being washed with distilled water, the sections were subjected to antigen retrieval in citrate buffer (pH 6.0) and stained overnight with rabbit polyclonal anti-BCOX1 antibodies (1∶200, Abcam, Cambridge, USA) at 4°C. After being washed with PBS (3×5 min), the sections were incubated with goat anti-rabbit IgG at room temperature for 30 min and developed with diaminobenzadine (DAB).

The relative levels of BCOX1 expression were evaluated semi-quantitatively, as described previously [10]. Briefly, the percentages and intensity of positive anti-BCOX1 staining in 10 high-power fields (magnification x400) selected randomly were evaluated by two investigators in blinded manner. The percentages of positively stained tumor cells in a field were scored as follows: 0: none; 1: <10%; 2∶10–50%; and 3: >50%. The staining intensity in a field was scored as follows: 0: no staining; 1: weak staining appearing as light yellow; 2: moderate staining appearing as yellowish-brown; and 3: strong staining appearing as brown. The staining index (SI) of individual sections was calculated as: (averaged staining intensity score)×(proportion score). The cut-off value for anti-BCOX1 staining was determined by measuring heterogeneity. Accordingly, an SI score of 4 (a cut-off value) was used to distinguish between low (<4) and high (≥4) levels of BCOX1 expression. Individual cases with discrepancies in the staining scores were re-reviewed by these two original pathologists together with a senior pathologist until a consensus was reached. Ultimately, the staining assessment and allocation of tumors by two investigators were similar with perfect inter-rater reliability (Kappa = 0.89).

### Statistical Analysis

Data are expressed as real case numbers or percentages. The difference between groups was analyzed by chi-square or Fisher exact test. The overall survival (OS) of the different groups of patients was estimated by the Kaplan-Meier survival curve and analyzed by the log-rank tests. The potential factors affecting the survival were analyzed by risk ratios (RRs), 95% confidence intervals (CI), univariate and multivariate regression analyses using the Cox proportional hazards model. All statistical analyses were performed using SPSS, version 11.0. A P value of <0.05 was considered statistically significant.

## Results

**Figure 1:**
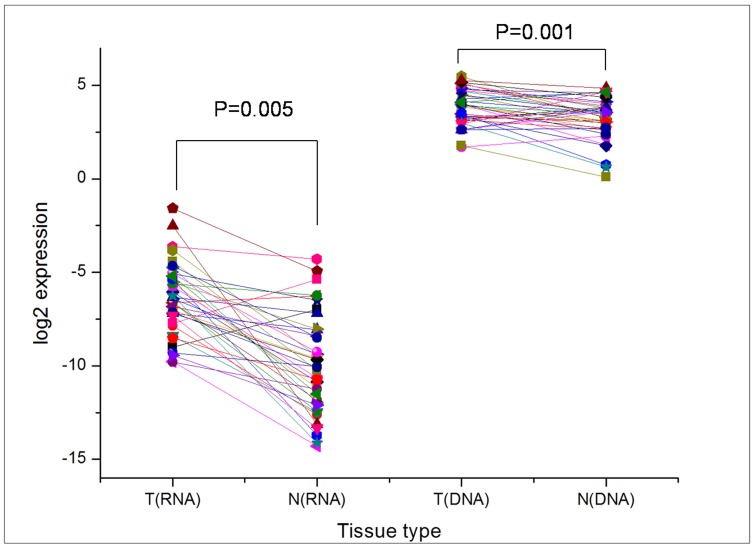
Quantitative RT-PCR analysis of the relative levels of BCOX1 mRNA transcripts. The relative levels of BCOX1 mRNA transcripts in 40 paired of breast cancer and surrounding non-tumor tissues were characterized by qRT-PCR suing specific primers. Briefly, total RNA was extracted from individual fresh frozen breast carcinomas and corresponding non-tumor breast tissues and reversely transcribed into cDNA, followed by qRT-PCR. The PCR amplification was performed in triplicate and the levels of BCOX1 mRNA transcripts were normalized against the control GAPDH. Data shown are individual values of the relative levels of BCOX1 mRNA transcripts for individual samples. T: Tumor tissues; N: the corresponding non-tumor tissues.

**Figure 2:**
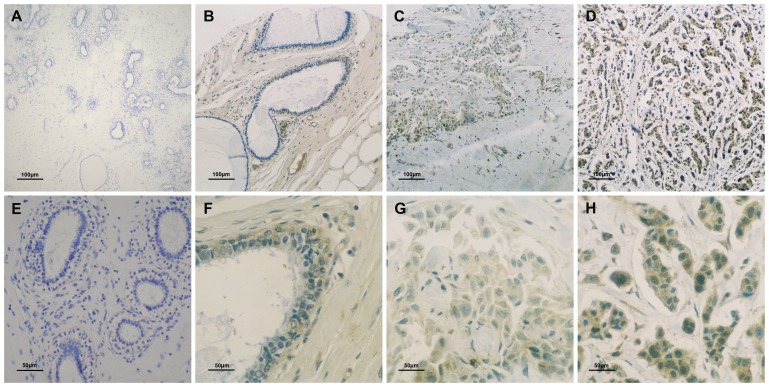
Immunohistochemistry analysis of BCOX1 expression. The BCOX expression in individual breast carcinoma tissue samples and the corresponding non-tumor tissues was characterized by tissue microarray and immunohistochemistry using specific antibody. Data shown are representative images (magnification×200 or 400) from 491 paired of IDC breast tissues. **A** and **E**: Negative staining for anti-BCOX1 in non-tumor breast tissues (**A**,×200; **E**,×400); **B** and **F**: Low levels of BCOX1 expression in non-tumor breast tissues (**B**,×200; **F**,×400); **C** and **G**: Low levels of BCOX1 expression in breast carcinoma tissues (**C**,×200; **G**,×400); **D** and **H**: High levels of BCOX1 expression in breast carcinoma tissues (**D**,×200; **H**,×400).

To determine the potential role of BCOX1 in the development and progression of IDC of the breast, 40 paired fresh tumor tissues and 491 paired IDC breast samples were collected and characterized for the relative levels of BCOX1 mRNA transcripts and protein expression by RT-PCR and immunohistochemistry. The demographic and clinical characteristics of these patients are summarized in Table 1. First, qRT-PCR analysis indicated that the relative levels of BCOX1 mRNA transcripts in 40 IDC breast tumor tissues were significantly higher than that in the corresponding non-tumor breast tissues (P = 0.005, Fig. 1). Immunohistochemistry analysis revealed that 292 (59.47%) out of 491 IDC breast tissues displayed a SI score of >4, an indicative of high BCOX1 expression and 199 (40.53%) of IDC breast tissues presented a SI score of <4, representing low BCOX1 expression (Fig. 2 and Table 2). Semi-quantitative analysis indicated that the levels of BCOX1 expression in 347 (70.7%) out of 491 IDC breast tissues were significantly higher than that in the corresponding non-tumor breast tissues, and 89 (18.1%) IDC breast tissues showed BCOX1 expression levels, similar to that in the corresponding non-tumor breast tissues. In contrast, the levels of BCOX1 expression in 55 (11.3%) IDC breast tissues were obviously lower than that in the corresponding non-tumor breast tissues.

**Table 1:** The demographic characteristics of study subjects.

Characteristics	No. of cases (%)
Age (years)	49 (25–78)
BCOX1 Negative	199 (40.53)
Positive	292 (59.47)
ER Negative	237 (48.27)
Positive	254 (51.73)
PR Negative	230 (46.84)
Positive	261 (53.16)
HER2 Negative	198 (40.33)
Positive	293 (59.67)
P53 Negative	337 (68.64)
Positive	154 (31.36)
Ki-67 Negative	305 (62.12)
Positive	186 (37.88)
Grade Negative	53 (10.79)
Positive	438 (89.21)
LNM Negative	94 (19.14)
Positive	397 (80.86)
Size Negative	192 (39.10)
Positive	299 (60.9)
Molecular subtype	
Luminal A	135 (27.49)
Luminal B	196 (39.92)
HER2	97 (19.76)
Triple negative	63 (12.83)

BCOX1, breast cancer overexpressed gene 1; ER, estrogen receptor; PR, progesterone receptor; HER2, human epidermal growth factor receptor 2; LNM, lymph node metastasis.

**Table 2:** Stratification analysis of the association of high BCOX1 expression with clinicopathological features.

			BCOX1		
Characteristics		No. ofcases	Negative (199)	Positive (292)	P
Age	<50	271	108	163	0.782
	≥50	220	91	129	
ER	Negative	237	106	131	0.080
	Positive	254	93	161	
PR	Negative	230	101	129	0.167
	Positive	261	98	163	
HER-2	Negative	198	92	106	**0.031**
	Positive	293	107	186	
P53	Negative	337	139	198	0.692
	Positive	154	60	94	
Ki-67	Negative	305	131	174	0.185
	Positive	186	68	118	
Grade	Negative	53	14	39	**0.037**
	Positive	438	185	253	
LNM	Negative	94	37	57	0.816
	Positive	397	162	235	
Size	Negative	192	84	108	0.259
	Positive	299	115	184	
Molecular subtype					
Luminal A		135	58	77	0.537
Luminal B		196	71	125	0.133
HER2		97	36	61	0.489
Triple negative		63	34	29	**0.027**

BCOX1, breast cancer overexpressed gene 1; ER, estrogen receptor; PR, progesterone receptor; HER2, human epidermal growth factor receptor 2; LNM, lymph node metastasis.

Stratification analysis revealed that there was no significant difference in the positive rates of high BCOX1 expression between patients with an age of <and >50 years old, and patients with negative or positive ER, PR, p53, Ki-67 expression (Table 2). Similarly, there was no significant difference in the positive rates of high BOCX1 expression between patients with or without lymph node metastasis, or with small and large tumor. However, the positive rates of high BCOX1 expression in patients with positive HER-2 expression were significantly higher than that in those with negative HER-2 breast cancer (P = 0.031). Similarly, the positive rates of high BCOX1 expression in patients with a high grade of breast cancer were significantly higher than those with low grade of tumor (P = 0.037). Moreover, the positive rates of high BCOX1 expression in triple negative breast cancer were significantly lower than that in the other molecular types of breast cancer. Therefore, high BCOX1 expression in breast cancer appeared to be significantly associated with HER-2 expression, high histological grade, and triple negative breast cancer.

**Figure 3:**
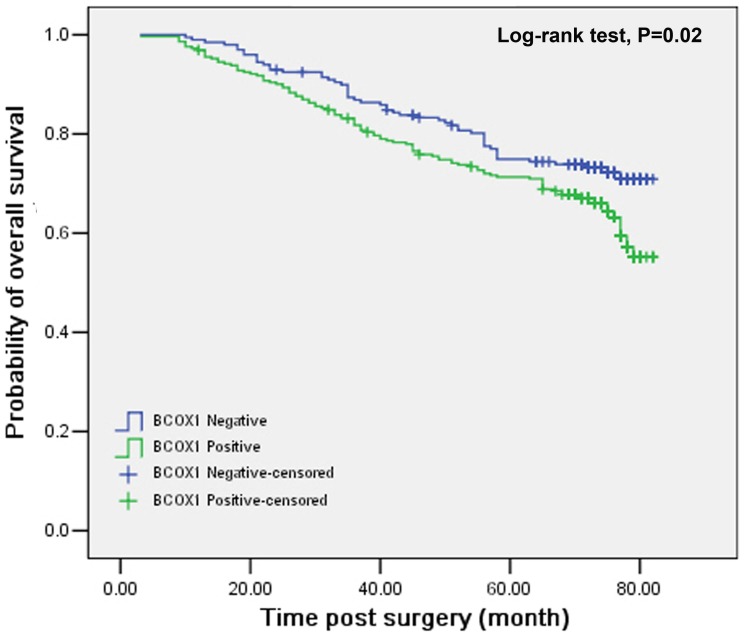
Stratification analysis of the association of positive BCOX1 expression with the overall survival in the patients with IDC of the breast. The patients with IDC of the breast were stratified, according to the positive or negative BCOX1 expression and their overall survival post surgery was estimated by the Kaplan-Meier method and analyzed by the log rank test. Data shown are the curves of overall survival of the different groups of patients. N = 289 for patients with positive BCOX1 expression; N = 199 for patients with negative BCOX1 expression.

**Figure 4:**
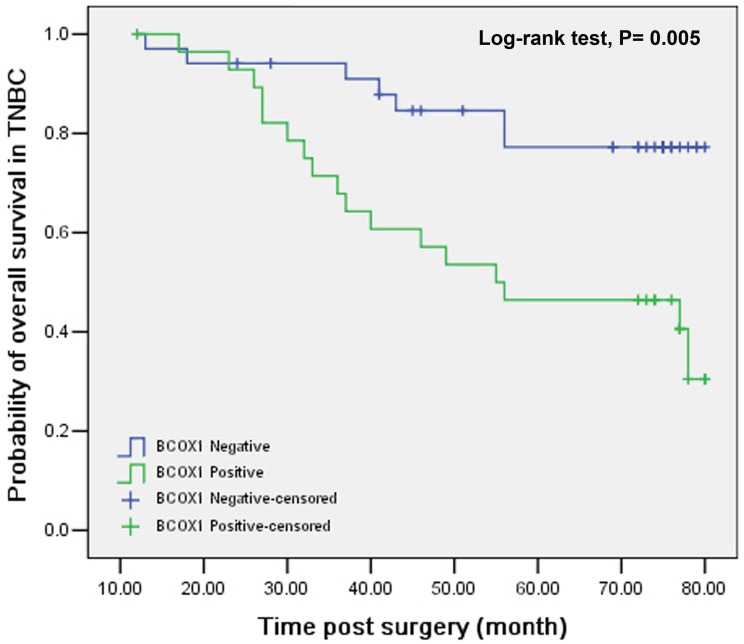
Stratification analysis of the association of positive BCOX1 expression with the overall survival in the patients with triple negative breast cancer. Patients with triple negative breast cancer were stratified, according to the positive or negative BCOX1 expression and their overall survival post surgery was estimated by the Kaplan-Meier method and analyzed by the log rank test. Data shown are the curves of overall survival of the different groups of patients. N = 29 for patients with positive BCOX1 expression; N = 34 for patients with negative BCOX1 expression.

To further evaluate the clinical significance of high BCOX1 expression, we evaluated the OS of patients with IDC of the breast and found that the survival period (66.10 months) of patients with positive BCOX1 expressing IDC of the breast were significantly shorter than that (70.43 months) in those with negative BCOX1 expressing tumors (P = 0.02, Fig. 3). Similarly, the survival period (55.84 months) of patients with positive BCOX1-expressing triple negative breast cancer were also significantly shorter than that (70.66 months) in those with negative BCOX1-expressing triple negative breast cancer (P = 0.005, Fig. 4). Finally, as shown in Table 3, univariate and multivariate survival analyses indicated that besides high histological grades (RR: 2.596, 95% CI: 1.276–5.284, P = 0.008 for univariate analysis and RR: 2.76, 95% CI: 1.362–5.658, P = 0.005 for mutiveriate analysis), high BCOX1 expression was an independent factor of the prognosis of patients with IDC of the breast in this population (RR: 1.453, 95% CI: 1.049–2.013, P = 0.025 for unveriate analysis and RR: 1.528, 95% CI: 1.102–2.117, P = 0.011 for mutiveriate analysis). Therefore, high BCOX1 expression was an independent prognostic factor for evaluating the survival of patients with IDC of the breast.

**Table 3:** Prognostic factors in the Cox model of proportional hazards.

Variables	Univariate			Mutivariate		
	RR	95% CI	P	RR	95% CI	P
Age (years)						
<50/≥50	1.006	0.740–1.368	0.970			
ER						
Negative/Positive	0.863	0.635–1.172	0.346			
PR						
Negative/Positive	0.795	0.585–1.080	0.142			
HER2						
Negative/Positive	0.898	0.659–1.223	0.494			
P53						
Negative/Positive	1.204	0.873–1.660	0.259			
Ki-67						
Negative/Positive	1.320	0.969–1.798	0.078			
Grade						
Negative/Positive	2.596	1.276–5.284	0.008	2.776	1.362–5.658	0.005
BCOX1						
Negative/Positive	1.453	1.049–2.013	0.025	1.528	1.102–2.117	0.011
LNM						
Negative/Positive	1.336	0.878–2.031	0.176			
Size						
Negative/Positive	1.160	0.843–1.596	0.361			

BCOX1, breast cancer overexpressed gene 1; RR, risk ratio; CI, confidence interval; ER, estrogen receptor; PR, progesterone receptor; HER2, human epidermal growth factor receptor 2; LNM, lymph node metastasis.

## Discussion

In this study, we found that the relative levels of BCOX1 mRNA transcripts in IDC breast tissues were significantly higher than that in the surrounding non-tumor tissues. The BCOX1 was predominantly expressed in the cytoplasm of breast tissue cells and high levels of BCOX1 expression were detected in 59.5% of 491 patients with IDC of the breast. Furthermore, we found that the high BCOX1 expression was associated significantly with HER-2 expression (P = 0.031) and high histological grade (P = 0.037) in patients with IDC of the breast. More importantly, high BCOX1 expression was associated with a shorter survival of patients with IDC of the breast, particularly for those with triple negative breast cancers. To the best of our knowledge, our findings provided the first evidence to demonstrate high levels of BCOX1 protein expression in breast cancer tissues and suggest that high levels of BCOX1 expression may be valuable for the prognosis of patients with IDC of the breast as well as a potential therapeutic target for intervention of IDC of breast in the clinic.

Breast tumorigenesis is a multi-step process starting from benign and atypical hyperproliferation, progressing into in situ carcinoma, invasive carcinomas, and culminating in metastatic disease [11]. The development and progression of breast cancer are involved in a complex process and attributed to the interaction of many genetic, epigenetic and environmental factors. Altered gene expression profiles may drive the disease progression. A previous study has stated that the BCOX1 mRNA transcripts are detected in breast cancer tissues, including moderate levels in ductal in situ carcinoma cells and high levels in invasive and metastatic breast cancer cells, but not in normal epithelial cells, stroma cells and lymphocytes [4]. However, they did show relative lower levels of BCOX1 mRNA transcripts in the corresponding normal breast tissues [4]. In this study, we detected relatively lower levels of BCOX1 mRNA transcripts in the cancer corresponding non-tumor tissues. Second, we found that although the levels of BCOX1 expression in the majority of IDC breast tissues were significantly higher than that in the surrounding non-tumor tissues the levels of BCOX1 expression in 29.4% of IDC breast tissues were either similar or significantly lower than that in the surrounding non-tumor tissues. Hence, it is possible that the BCOX1 may be expressed in breast cancer surrounding non-tumor tissues. Currently, we have no definite explanation on the discrepancy. Possibly, the discrepancy between our and their observations may stem from different populations of patients with varying types of breast cancers and the different methods for preparing samples. Given that the prepared tissue lysates for qRT-PCR may contain variable epithelial cellularity and potential contamination of a few tumor cells. Accordingly, the qPCR data from lysates of normal breast tissues might be of limited value in assessing epithelial BCOX1 mRNA transcripts as the epithelial cellularity in the tissue lysates might be variable. We are interested in further determining the expression profiles of BCOX1 in breast cancer tissues.

Our study showed that the high BCOX1 expression was associated significantly with high HER2 expression (P = 0.031) in IDC breast tissues. Further stratification analyses indicated that there were 97 out of 293 high HER2 expressing IDC breast samples with the HER2 gene amplification. However, there was no significant association between the high BCOX1 expression and HER2 gene amplification in this population. Previous studies have shown that high levels of HER2 expression in breast cancer tissues are significantly associated with a poor prognosis of patients with breast cancer [12], [13]. In this study, we found that the high BCOX1 expression was significantly associated with a shorter survival of patients with IDC of the breast. Univariate and multivariate analyses indicated that high BCOX1 expression was an independent factor of the poor prognosis of patients with IDC of the breast in this population. These findings suggest that high BCOX1 expression may promote the recurrence of IDC of the breast. However, it remains to determine whether high BCOX1 and HER2 expression can synergistically promote the progression of breast cancer.

Interestingly, we found that high BCOX1 expression was associated with triple negative breast cancer, which is a unique cellular type of breast cancer with aggressively biological behaviors and poor clinical outcome [14]. Currently, there is no effective therapy for patients with triple negative breast cancer [15], [16]. More importantly, we found that high BCOX1 expression was associated significantly with a shorter survival of patients with triple negative breast cancer. It is possible that high BCOX1 expression may also promote the recurrence and progression of triple negative breast cancer. Apparently, high BCOX1 expression may be a valuable prognostic marker for evaluating the survival of patients with triple negative breast cancer.

We recognized that our study had limitations. First, the relatively small sample size in a single center may limit the statistical power. Second, this study only included Chinese while there was ethic difference in the prognosis of patients with triple negative breast cancer [17], [18]. Furthermore, we did not characterize the copy numbers and polymorphism of the BCOX1 gene in these subjects, which might affect the value of high BCOX1 expression in the prognosis of patients IDC of the breast because high BCOX1 expression may be a marker of a nearby gene of more biological significance in breast cancer. In addition, we did not test the functional role of high BCOX1 expression in the development and progression of breast cancer. Further studies in a bigger population with multi-ethnicity in multi-centers to validate these findings and to explore the functional role and mechanisms are warranted.

In conclusion, our data from this study showed that high levels of BCOX1 were expressed in the majority of IDC breast tissues and associated with high HER2 expression and high histological grade of breast cancer. High BCOX1 expression was associated with significantly with a poor prognosis of patients with IDC of the breast, particularly for those with triple negative breast cancer. Therefore, the high BCOX1 expression may serve as a molecular marker for evaluating the prognosis and a therapeutic target for the intervention of patients with IDC of the breast. Further understanding the function and molecular mechanisms of BCOX1 in regulating the progression of breast cancer may provide new insights into breast tumorigenesis.

## References

[1] Siegel R, Naishadham D, Jemal A (2013) Cancer statistics, 2013. CA Cancer J Clin 63: 11–30. 23335087 10.3322/caac.21166

[2] Wang Q, Zhao ZB, Wang G, Hui Z, Wang MH, et al (2013) High Expression of KIF26B in Breast Cancer Associates with Poor Prognosis. PLoS One 8: e61640. 23585914 10.1371/journal.pone.0061640 PMC3621833

[3] Mishra DK, Wu Y, Sarkissyan M, Sarkissyan S, Chen Z, et al (2013) Vitamin D Receptor Gene Polymorphisms and Prognosis of Breast Cancer among African-American and Hispanic Women. PLoS One 8: e57967. 23554871 10.1371/journal.pone.0057967 PMC3595235

[4] Song J, Yang W, Shih Ie M, Zhang Z, Bai J (2006) Identification of BCOX1, a novel gene overexpressed in breast cancer. Biochim Biophys Acta 1760: 62–69. 16289875 10.1016/j.bbagen.2005.09.017

[5] Nagase T, Miyajima N, Tanaka A, Sazuka T, Seki N, et al (1995) Prediction of the coding sequences of unidentified human genes. III. The coding sequences of 40 new genes (KIAA0081–KIAA0120) deduced by analysis of cDNA clones from human cell line KG-1 (supplement). DNA Res 2: 51–59. 7788529 10.1093/dnares/2.1.51

[6] Cheang MC, Chia SK, Voduc D, Gao D, Leung S, et al (2009) Ki67 index, HER2 status, and prognosis of patients with luminal B breast cancer. J Natl Cancer Inst 101: 736–750. 19436038 10.1093/jnci/djp082 PMC2684553

[7] Aksoy S, Dizdar O, Harputluoglu H, Altundag K (2007) Demographic, clinical, and pathological characteristics of Turkish triple-negative breast cancer patients: single center experience. Ann Oncol 18: 1904–1906. 17993632 10.1093/annonc/mdm487

[8] Bauer KR, Brown M, Cress RD, Parise CA, Caggiano V (2007) Descriptive analysis of estrogen receptor (ER)-negative, progesterone receptor (PR)-negative, and HER2-negative invasive breast cancer, the so-called triple-negative phenotype: a population-based study from the California cancer Registry. Cancer 109: 1721–1728. 17387718 10.1002/cncr.22618

[9] Sorlie T, Perou CM, Tibshirani R, Aas T, Geisler S, et al (2001) Gene expression patterns of breast carcinomas distinguish tumor subclasses with clinical implications. Proc Natl Acad Sci U S A 98: 10869–10874. 11553815 10.1073/pnas.191367098 PMC58566

[10] Liu T, Zhang X, Shang M, Zhang Y, Xia B, et al (2013) Dysregulated expression of Slug, vimentin, and E-cadherin correlates with poor clinical outcome in patients with basal-like breast cancer. J Surg Oncol 107: 188–194. 22886823 10.1002/jso.23240

[11] Beckmann MW, Niederacher D, Schnurch HG, Gusterson BA, Bender HG (1997) Multistep carcinogenesis of breast cancer and tumour heterogeneity. J Mol Med (Berl) 75: 429–439. 9231883 10.1007/s001090050128

[12] Yarden Y (2001) Biology of HER2 and its importance in breast cancer. Oncology 61 Suppl 21–13. 10.1159/000055396 11694782

[13] Ang KK, Berkey BA, Tu X, Zhang HZ, Katz R, et al (2002) Impact of epidermal growth factor receptor expression on survival and pattern of relapse in patients with advanced head and neck carcinoma. Cancer Res 62: 7350–7356. 12499279

[14] Reis-Filho JS, Tutt AN (2008) Triple negative tumours: a critical review. Histopathology 52: 108–118. 18171422 10.1111/j.1365-2559.2007.02889.x

[15] Anders CK, Carey LA (2009) Biology, metastatic patterns, and treatment of patients with triple-negative breast cancer. Clin Breast Cancer 9 Suppl 2S73–81. 19596646 10.3816/CBC.2009.s.008 PMC2919761

[16] Ma KK, Chau WW, Wong CH, Wong K, Fung N, et al (2012) Triple negative status is a poor prognostic indicator in Chinese women with breast cancer: a ten year review. Asian Pac J Cancer Prev 13: 2109–2114. 22901178 10.7314/apjcp.2012.13.5.2109

[17] Morris GJ, Naidu S, Topham AK, Guiles F, Xu Y, et al (2007) Differences in breast carcinoma characteristics in newly diagnosed African-American and Caucasian patients: a single-institution compilation compared with the National Cancer Institute’s Surveillance, Epidemiology, and End Results database. Cancer 110: 876–884. 17620276 10.1002/cncr.22836

[18] Amirikia KC, Mills P, Bush J, Newman LA (2011) Higher population-based incidence rates of triple-negative breast cancer among young African-American women: Implications for breast cancer screening recommendations. Cancer 117: 2747–2753. 21656753 10.1002/cncr.25862 PMC3461243

